# Supplementary material for: Anti-Acetylcholinesterase Activities of Mono-Herbal Extracts and Exhibited Synergistic Effects of the Phytoconstituents: A Biochemical and Computational Study
Source: Molecules. 2019 Nov 18;24(22):4175. doi: 10.3390/molecules24224175 (PMC6891289; doi:10.3390/molecules24224175)
Supplement: Supplementary file 1 [file molecules-24-04175-s001.pdf]

-Supplementary Materials-

Anti-acetylcholinesterase activities of mono-herbal extracts and exhibited synergistic effects of the phytoconstituents: a biochemical and computational study

(A). Materials and Methods (HPLC analysis)

Test Article:

T. cordifolia (Giloy)- methanolic extract

Sample preparation method:

10 ml methanol was added in 0.5 gm extract and sonicated for 30 min, centrifuged at 8000 rpm for 5 min, and filtered using 0.45 µm nylon filter. This filtered solution was used for the analysis of palmatine, berberine, gallic acid, vanillic acid and ferulic acid.

Standard preparation method:

Standards were purchased from Natural Remedies Pvt. Ltd. and Sigma Aldrich, Bangalore, India. Details of the standards are given in below in Table

Standards used for analysis

S. N.	Name	Manufacturer/ Supplier
**1**	Gallic Acid	Sigma Aldrich
**2**	Palmetin	Natural Remedies
**3**	Berberine	Natural Remedies
**4**	Venillic Acid	Sigma Aldrich
**5**	Ferulic Acid	Natural Remedies

All the standards were dissolved in methanol to prepare the appropriate concentrations.

Experimental Methods:

The quantification of marker compounds was performed by waters HPLC equipped with binary pump (1525), PDAD (2998) & auto-sampler (2707). The elution was carried out at a flow rate of 1.0 ml/min using gradient elution of mobile phase A (0.140 gm of KH_2PO_4 dissolved in 1000 ml of water, adjust pH 2.5 with orthophosphoric acid) and mobile phase B (acetonitrile). This experiment was performed on Shodex C18-4E (4.6mm ID x 250 mm L) column and column temperature was kept at 35°C during the analysis. 10 µl of test solution was injected during the analysis and recorded the chromatographs at 346 and 270 nm wavelengths.

Gradient Program:

Time (Min)	Flow (ml/min)	A %	B%	Curve
**0**	1.0	95	5	6
**3**	1.0	95	5	6
**10**	1.0	85	15	6
**15**	1.0	80	20	6
**20**	1.0	70	30	6
**25**	1.0	65	35	6
**30**	1.0	65	35	6
**35**	1.0	10	90	6
**40**	1.0	10	90	6
**41**	1.0	95	5	6
**45**	1.0	95	5	6

(B). Results

LOD and LOQ:

LOD and LOQ of marker compounds

Marker Compound	LOD	LOQ
Gallic Acid	0.5 mcg/ml	1.0 mcg/ml
Palmetin	0.5 mcg/ml	1.0 mcg/ml
Berberine	0.5 mcg/ml	1.0 mcg/ml
Venillic Acid	0.5 mcg/ml	1.0 mcg/ml
Ferulic acid	0.5 mcg/ml	1.0 mcg/ml

Linearity plots:

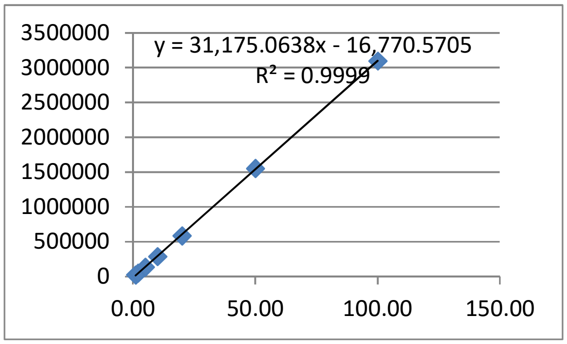

Linearity plot of gallic acid

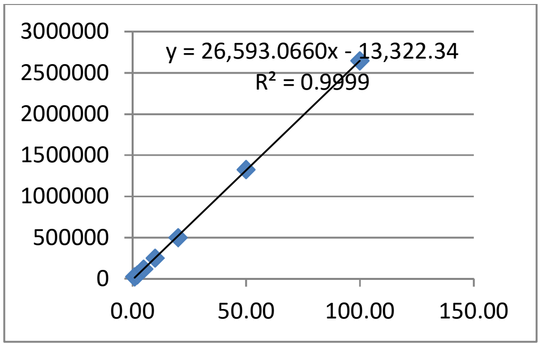

Linearity plot of vanillic acid

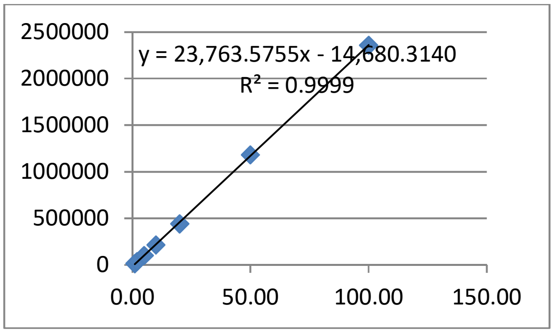

Linearity plot of ferulic acid

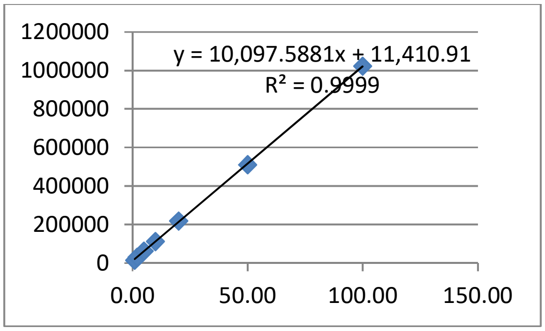

Linearity plot of palmatine

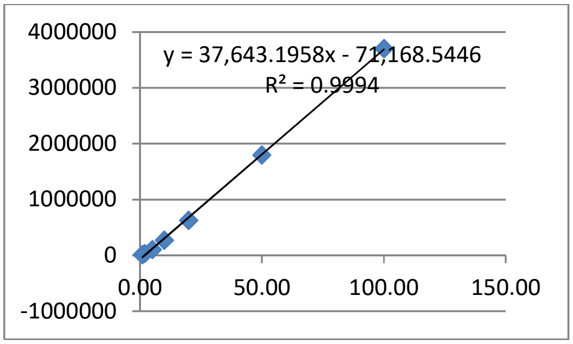

Linearity plot of berberine

Regression equation and correlation coefficient for marker compounds

Name Of Marker Compound	Regression Equation	Correlation Coefficient
**Gallic Acid**	y= 31,175.06x - 16,770.57	0.9999
**Palmatine**	y= 10,097.58x + 11,410.91	0.9999
**Berberine**	y = 37,643.19x - 71,168.54	0.9994
**Vanillic acid**	y= 26,593.06x - 13,322.34	0.9999
**Ferulic acid**	y = 23,763.57x - 14,680.31	0.9999

Precision:

Intraday and interday precision of marker compounds

	Gallic Acid (at 270 nm)	Palmatine (at 346 nm)	Berberine (at 346 nm)	Vanillic acid (at 270 nm)	Ferulic acid (at 346 nm)
**Intra Day**	**Result in mg/gm**	**Result in mg/gm**	**Result in mg/gm**	**Result in mg/gm**	**Result in mg/gm**
**1**	0.136	0.147	0.023	0.511	0.204
**2**	0.131	0.150	0.023	0.508	0.201
**3**	0.134	0.153	0.022	0.511	0.211
**4**	0.132	0.154	0.022	0.502	0.202
**5**	0.132	0.149	0.022	0.495	0.197
**6**	0.131	0.148	0.022	0.500	0.197
**Average**	0.133	0.150	0.022	0.504	0.202
**Stdev**	0.002	0.003	0.003	0.006	0.005
**% RSD**	**1.54**	**1.86**	**2.86**	**1.26**	**2.53**
**Inter Day**					
**1**	0.135	0.150	0.023	0.511	0.205
**2**	0.133	0.157	0.022	0.489	0.199
**3**	0.134	0.161	0.022	0.494	0.199
**4**	0.135	0.160	0.022	0.492	0.199
**5**	0.134	0.164	0.022	0.492	0.199
**6**	0.131	0.160	0.022	0.483	0.197
**Average**	0.134	0.159	0.022	0.494	0.200
**Stdev**	0.002	0.005	0.001	0.009	0.003
**% RSD**	**1.28**	**2.94**	**2.27**	**1.91**	**1.39**

Accuracy (Recovery):

Recovery of marker compounds

S.No	Compound Name	% Recovery Level 1	% Recovery Level 2	% Recovery Level 3
**1**	Gallic Acid	98.90	98.75	97.08
**2**	Palmatine	98.07	99.00	99.29
**3**	Berberine	98.32	97.00	96.30
**4**	Vanillic acid	98.33	99.40	98.50
**5**	Ferulic acid	97.42	99.35	96.75

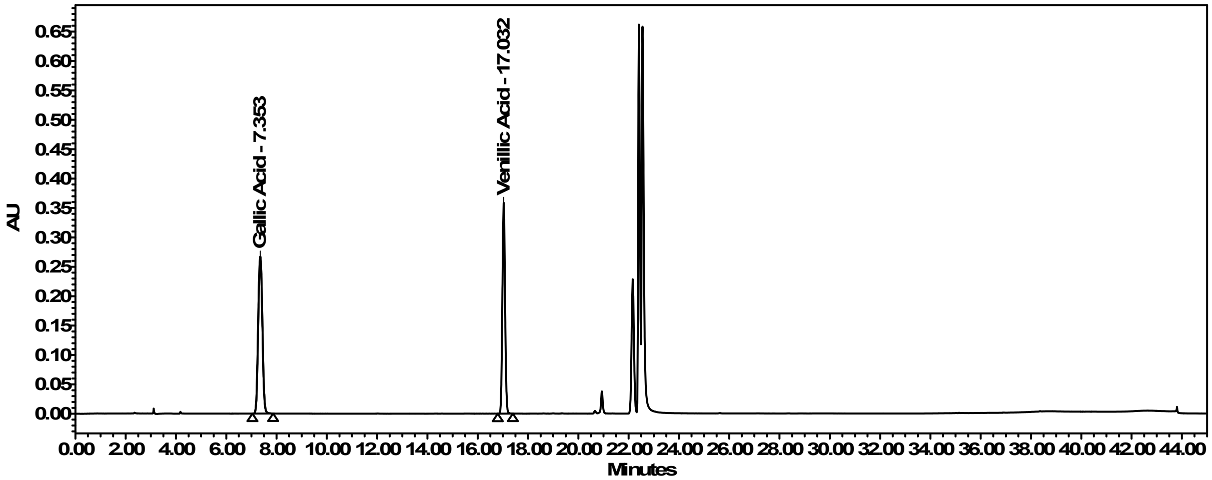

Image 1: Standard mix chromatographs of gallic acid (7.353 min) and vanillic acid (17.032 min).

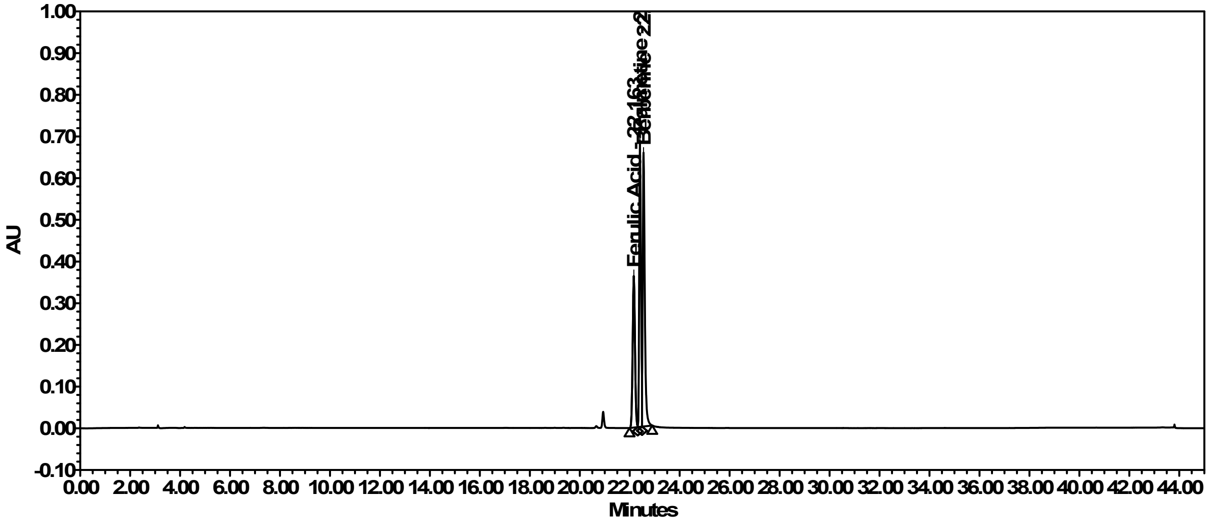

Image 2: Standard mix chromatographs of ferulic acid (22.163 min), palmatine (22.407 min) and berberine (22.553 min).

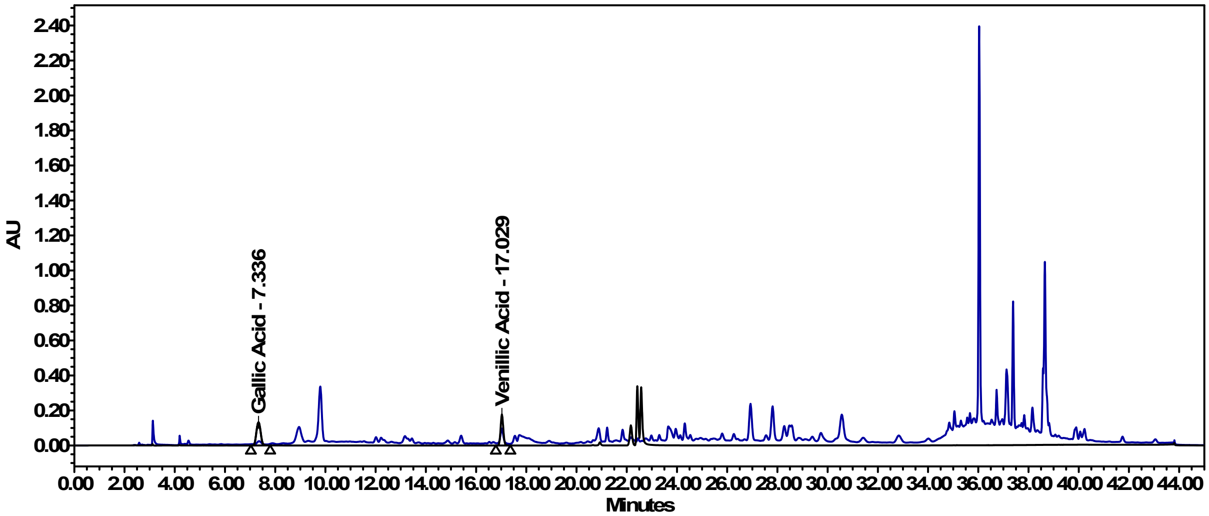

Image 3: Overlap chromatograph of standard mix gallic acid, vanillic acid and Giloy methanolic extract at 270 nm.

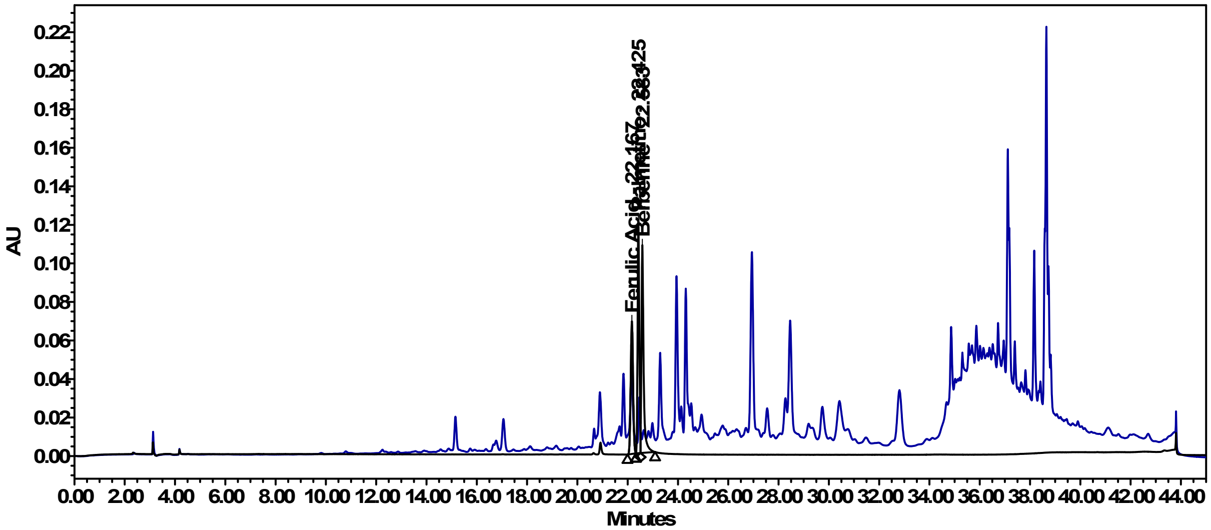

Image 4: Overlap chromatograph of standard mix of ferulic acid, palmatine, berberine and Giloy methanolic extract at 346 nm.

Result :

Result of marker compounds in Giloy - methanolic extract

S.No.	Name Of Marker Compound	Results in mg/gm
1	Gallic Acid	**0.134**
2	Palmatine	**0.159**
3	Berberine	**0.022**
4	Vanillic acid	**0.494**
5	Ferulic acid	**0.205**

Conclusion:

HPLC method was developed and validated for the analysis of Giloy - methanolic extract using five marker compounds. It was found that 0.134 mg/gm gallic acid, 0.159 mg/gm palmatine, 0.022 mg/gm berberine, 0.494 mg/gm vanillic acid and 0.205 mg/gm ferulic acid were present in Giloy methanolic extract.
